# Caregiving during the Covid-19 pandemic: Factors associated with caregiver stress and cognition

**DOI:** 10.1093/geroni/igab046.2950

**Published:** 2021-12-17

**Authors:** Rachael Turner, Erin Harrington, Celinda Reese-Melancon

**Affiliations:** 1 Oklahoma State University, Stillwater, Oklahoma, United States; 2 Oklahoma State University, Oklahoma State University, Oklahoma, United States

## Abstract

Caregivers are critical in helping persons with dementia (PWD) live at home longer, but the caregiving experience is associated with increased risk of physical (Vitaliano et al., 2003; Son et al., 2007; Fonareva & Oken, 2014) and cognitive decline among caregivers (Pertle et al., 2015; Lathan et al., 2016; Vitaliano et al., 2017). The present study examined the caregiver experience during the time of the Covid-19 pandemic including factors associated with caregiver stress, burden, and self-reported cognition (i.e., prospective and retrospective memory errors). In a sample of 56 caregivers of PWD, caregiver stress was positively associated with reports of greater life change resulting from Covid-19 and a greater frequency of care recipient depressive and disruptive behaviors; however, caregiver stress was not associated with care recipient memory problems. Additionally, caregiver burden was negatively associated with ratings of preparedness for the pandemic, but not with availability of support services or the amount of time spent caregiving. Further, frequencies of prospective and retrospective memory mistakes were positively associated with perceived stress, but not with caregiver burden. These findings reveal that caregivers of PWD report greater experiences of stress associated with the Covid-19 pandemic and other facets of their caregiving responsibilities (e.g., care recipient depressive and disruptive behaviors, frequency of memory mistakes). This work is a first step in identifying areas in which caregivers need assistance and expanding the literature on caregiver cognition by measuring self-reported everyday memory performance.

